# Evaluation of the Mean Platelet Volume and Red Cell Distribution Width in FMF: Are They Related to Subclinical Inflammation or Not?

**DOI:** 10.1155/2014/127426

**Published:** 2014-03-12

**Authors:** Gozde Yildirim Cetin, Ozlem Gul, Fatma Kesici-Metin, İrem Gokalp, Mehmet Sayarlıoglu

**Affiliations:** ^1^Division of Rheumatology, Department of Internal Medicine, School of Medicine, Faculty of Medicine, Sutcu Imam University, 46050 Kahramanmaras, Turkey; ^2^Division of Hematology, Department of Pediatrics, School of Medicine, Sutcu Imam University, 46050 Kahramanmaras, Turkey; ^3^Department of Internal Medicine, School of Medicine, Sutcu Imam University, 46050 Kahramanmaras, Turkey; ^4^Division of Rheumatology, Department of Internal Medicine, School of Medicine, Ondokuz Mayis University, 55000 Samsun, Turkey

## Abstract

In this paper we want to demonstrate whether higher than normal levels of RDW, and lower than normal levels of MPV can be used as indicators of subclinical inflammation and tools for treatment decision in FMF or not. The participants in this study included 89 patients with FMF during attack-free periods and 30 healthy controls. The RDW and platelet counts were significantly higher, while the MPV was significantly lower in the patients with FMF group than healthy control group (*P* < 0.001; *P* = 0.005; *P* < 0.001, resp.). In the attack-free FMF group, a negative correlation was found between the MPV and RDW values (*P* < 0.001, *r* = −0.40). The positive correlation was found between the RDW and ESR (*r* = 0.23, *P* = 0.028). And the negative correlation was found between the MPV and CRP (*r* = −0.216, *P* = 0.042). Consequently, our results suggest that low MPV and high RDW levels may provide additional information about subclinical inflammation in FMF patients. But other strong predisposing factors affecting subclinical inflammation in FMF should be considered. Further studies with large numbers of patients are needed. Treatment of FMF should include not only prevention of acute attacks but also decreasing of the subclinical inflammation.

## 1. Introduction

Familial Mediterranean fever (FMF) is characterized by recurrent and self-limiting attacks with peritonitis, pleuritis, arthritis, and erysipelas-like erythema. It is inherited as an autosomal recessive trait and prominently presents among Sephardi Jews, Turks, Armenians, Arabs, and, to a much lesser extent, other ethnicities [1–3]. The attacks of FMF are only the tip of the iceberg, and inflammation maintains in attack-free remission periods in 30% of patients with FMF [4]. This maintaining subclinical inflammation induces endothelial dysfunction and increases the risk of developing significant complications such as atherothrombosis, anemia, splenomegaly, decreased bone mineral density, heart disease, and life-threatening secondary systemic amyloidosis [5, 6]. Subclinical inflammation is shown by high levels of acute-phase proteins, cytokines, and inflammation-induced proteins (e.g., overproduction of C-reactive protein (CRP) or serum amyloid-A (SAA) and persistently elevated fibrinogen levels and erythrocyte sedimentation rates (ESR)) in attack-free FMF periods [5, 7–9].

Red cell distribution width (RDW) is commonly accepted as part of the hemogram and refers to the changeability in the size of the erythrocytes in the peripheral circulation. RDW is used to refer to the differential diagnosis of anemia, and it increases during inflammation; therefore, RDW can increase as a result of chronic inflammation [10, 11].

Platelet volume, a marker of platelet function and activation, is generally measured as the mean platelet volume (MPV) [12]. Low MPV levels can be present in high-grade inflammatory diseases such as active RA and AS or attacks of FMF. This has been demonstrated in previous studies [13–16]. An increased MPV was found to be negatively correlated with inflammation in patients with ulcerative colitis and Crohn's disease [17], and, in a recent study, it was shown that anti-TNF-alpha therapy decreases the MPV in AS patients [18].

In this paper we want to demonstrate whether higher than normal levels of RDW and lower than normal levels of MPV can be used as indicators of subclinical inflammation and tools for treatment decision in FMF or not.

## 2. Materials and Methods

In this study, 89 patients with FMF (male/female: 30/59, mean age: 31.03 ± 10.2/32.18 ± 10) and 30 age- and sex-matched healthy controls (male/female: 10/20, mean age: 32.9 ± 6.5/30.65 ± 5.2) were enrolled. The patient group was comprised of 89 patients who fulfilled clinical diagnostic criteria for FMF. All patients were under colchicine treatment and they were in an attack-free period. All of them were responders to colchicine with ≤1 FMF attack within the six months or without any attacks over the twelve months. The ESR, CRP, white blood cell count, platelet count, hemoglobin, MPV, and RDW levels were retrospectively recorded. These patients were evaluated in accordance with the “Disease Severity Score,” the Tel-Hashomer severity scoring, which includes six elements: disease onset age, dose of colchicine, number of involved sites in one attack and during the course of the disease, and the presence of pleuritic and erysipelas-like erythema.

## 3. Statistical Analysis

Student's *t*-test and one-way ANOVA were used for comparison of averages. Pearson's correlation analyses were performed to evaluate other factors. *P* value of <0.05 was accepted as statistically significant.

## 4. Results

The demographic features and laboratory results of attack-free patients with FMF and the control healthy groups are shown in Table 1. Mean age of disease onset was 19.4 ± 9.66 (min: 1 and max: 44) years. Median colchicine dose was 1.50 (min: 1 and max ≥ 1.5) mg/day. The mean age, male/female ratio, mean levels of hemoglobin, and white blood cell count were similar in the two groups. Two of 89 (2.24%) patients with FMF had amyloidosis in our study. The RDW and platelet counts were significantly higher and the MPV was significantly lower in the patients with FMF group (15.59 ± 1.6% versus 12.9 ± 0.8%, *P* < 0.001; 289 ± 78.2 K/*μ*L versus 245.63 ± 55.7 K/*μ*L, *P* = 0.005; and 7.9 ± 1 fL versus 9.4 ± 0.9 fL, *P* < 0.001, resp.). All parameters excluding hemoglobin were similar for males and females. Hemoglobin counts were significantly higher in male group (13.01 ± 1.06 versus 14.75 ± 1.18, *P* = 0.00) (Table 2). However hemoglobin counts were in normal range in both of the two groups. In the attack-free FMF group, a negative correlation was found between the MPV and RDW values (*P* < 0.001, *r* = −0.40). Median disease severity score was 4.6 (min: 2 and max: 13), where 69 cases (77.5%) were scored as “mild,” 16 cases (17.9%) were “moderate,” and 4 cases (4.49%) were “severe.” The correlation was not found between the disease severity score and MPV, RDW, ESR, and CRP values (*P* = 0.82, *r* = −0.024; *P* = 0.75, *r* = −0.033; *P* = 0.24,  *r* = 0.12; and *P* = 0.57, *r* = −0.061, resp.). However, the positive correlation was found between the RDW and ESR (*r* = 0.23, *P* = 0.028). And the negative correlation was found between the MPV and CRP (*r* = −0.216,  *P* = 0.042).

## 5. Discussion

In our study we found the positive correlation between the RDW and ESR and the negative correlation between the MPV and CRP in attack-free FMF period. Förhécz et al. showed that CRP had a significantly positive correlation with RDW. It was found that, between prealbumin, which is the negative acute-phase reactant, and RDW, a significantly negative correlation exists in patients with heart failure [11]. In a recent study, Lippi et al. showed a correlation of RDW with CRP and ESR in unselected adult outpatients [19], while Erdem et al. demonstrated that patients with an inflammatory condition, such as reactive systemic AA amyloidosis, have higher RDWs and lower MPVs [20].

In our study the RDW and platelet counts were significantly higher and the MPV was significantly lower in the patients with attack-free FMF group than healthy control group. Subclinical inflammation is observed during the course of FMF and diagnosed by measuring cytokine levels including interleukin (IL)-6, IL-8, IL-12, IL-17, IL-18, interferon, and tumor necrosis factor, which have been shown to increase during and between the attacks [21–28]. IL-6 is one of the proinflammatory cytokines that can cause thrombocytosis and change platelet volume. It has been shown to increase the platelet number and decrease the MPV in patients with cancer [29]. So the higher RDW and lower MPV can be observed in chronic inflammation such as attack-free FMF patients.

In our study we did not find correlation between the “Disease Severity Score” and MPV, RDW, ESR, and CRP values. This can be explained with other factors that can affect severity of attacks and ongoing subclinical inflammation in FMF. Patients with phenotype II FMF without ever having an acute, painful, febrile attack develop secondary amyloidosis because of subclinical inflammation. A maintained, long-term elevation in SAA levels is prerequisite for occurring of secondary amyloidosis [30, 31]. Levels of both CRP and SAA are increased in 30–90% of patients with FMF during attack-free period, even while using colchicine therapy. Levels of these proteins are also increased in asymptomatic carriers of MEFV mutations which means that these apparently unaffected individuals have ongoing subclinical inflammation [5, 32].

On the other hand colchicine therapy affects strongly subclinical inflammation in FMF. Other factors such as male sex, age of disease onset, frequency of acute attacks, joint involvement during attacks, risk factors for the development of amyloidosis, and markers of inflammation are related to chronic inflammation in FMF [33–35]. As a result of our study increasing of RDW and decreasing of MPV levels not only can occur with severe chronic inflammation and could be useful to predict who will develop amyloidosis but also other strong predisposing factors should be considered.

The acute attacks of FMF are self-limited. Treatment is purposed of reprieve reducing pain with analgesics, NSAIDs, and rest during attacks. Colchicine does not suppress acute attack. The aim of colchicine therapy is to prevent acute attacks and amyloidosis [36–38]. Colchicine therapy is started as an oral dose of 1.0–1.2 mg daily and increased as needed to control attacks until a maximum dose of 2.0–2.4 mg daily is reached [6]. Colchicine therapy reduces levels of the markers of subclinical inflammation such as ESR, CRP, and white blood cell count in asymptomatic patients with FMF. These markers can be used for following colchicine response [39]. SAA level decreases with increased colchicine dose in attack-free patients with FMF [40]. Our study supports that RDW and MPV can be used like these markers. If patient's adherence is bad or response to treatment is poor despite sufficient dose, colchicine dose should be raised to 2.5–3.0 mg daily or add a weekly dose (1 mg) of intravenous colchicine or even try treatment with subcutaneous interferon-alpha [41].

## 6. Conclusions

Consequently, our results suggest that low MPV and high RDW levels may provide additional information about persistent subclinical inflammation in FMF patients during attack-free periods. But other strong predisposing factors affecting subclinical inflammation in FMF should be considered. Further studies with large numbers of patients are needed.Treatment of FMF should include not only prevention of acute attacks but also decreasing of the chronic subclinical inflammation.

## Figures and Tables

**Table 1:** Attack-free patients with FMF and healthy control groups' demographics and comparison of laboratory parameters.

	Group of attack-free patients with FMF (*n* = 89) Mean ± SD	Healthy control group (*n* = 30) Mean ± SD	*P* value
Age (years)	31.8 ± 10	31.4 ± 5.7	0.83
Gender (M/F)	30/59	10/20	1
Hemoglobin (g/dL)	13.4 ± 1.38	14.02 ± 1.26	0.05
MPV (fL)	7.9 ± 1	9.4 ± 0.9	**<0.001 **
RDW (%)	15.59 ± 1.6	12.9 ± 0.8	**<0.001 **
Platelet count (10^3^/mm^3^)	289 ± 78.2	245.63 ± 55.7	**0.005 **
White blood cell (/mm^3^)	7116.2 ± 1737	7183 ± 2075	0.86

ESR: erythrocyte sedimentation rate; F: female; M: male; MPV: mean platelet volume; RDW: red cell distribution width; SD: standard deviation.

**Table 2:** The demographics and comparison of laboratory parameters for males and females.

	Gender	*N*	Mean	Std. deviation	*P* value
Age	F M	70 40	31.79 31.50	9.06 9.39	0.86

WBC	F M	79 40	7081.27 7232.98	1682.840 2072.126	0.67

Plt	F M	79 40	284.00 267.78	72.179 81.585	0.27

RDW	F M	79 40	15.0671 14.5620	1.94176 1.43472	0.14

MPV	F M	79 40	8.4051 8.1080	1.28828 1.11365	0.21

Hbg	F M	79 40	13.0139 14.7568	1.06334 1.18326	**0.00 **

F: female; M: male; MPV: mean platelet volume (fL); RDW: red cell distribution width (%); WBC: white blood cell (/mm^3^); Plt: platelet count (10^3^/mm^3^); Hbg: hemoglobin (g/dL).
